# Structural and functional modulation of *Fagopyrum tataricum* by multifrequency ultrasonication, germination and soaking: impact on polyphenols and techno-functional properties^☆^

**DOI:** 10.1016/j.ultsonch.2026.107994

**Published:** 2026-08-05

**Authors:** Zunaira Basharat, Bin Dai, Huicong Xu, JiaYu Yin, Aysha Imtiaz, Muhammad Safiullah Virk, Sanabil Yaqoob, Yuqing Duan, Kai Hu, Meihong Cai, Haihui Zhang, Qing Shen, Chunlai Zeng

**Affiliations:** a School of Food Science and Engineering, Jiangsu University, Zhenjiang 212013, China; b Institute of Food Physical Processing, Jiangsu University, Zhenjiang 212013, China; c School of Fisheries and Life Sciences, Shanghai Ocean University, Shanghai 201306, China; d College of Life Sciences, Guizhou University, Guiyang 550025, China; e Laboratory of Food Nutrition and Clinical Research, Institute of Seafood, Zhejiang Gongshang University, Hangzhou 310012, China; f Department of Cardiology, Central Laboratory of Lishui Hospital of Wenzhou Medical University, The First Affiliated Hospital of Lishui University, Lishui People’s Hospital, Lishui, Zhejiang 323000, China

**Keywords:** *Fagopyrum tataricum*, Multifrequency ultrasonication, Germination, Polyphenols, Volatile compounds, Techno-functional properties

## Abstract

The current study investigated the effect of multifrequency ultrasonication (MFUT), germination (G), soaking (S) and their combined effect treatments MFUT + G and MFUT + G + S on metabolite profile, structural modifications and techno-functional properties of *Fagopyrum tataricum.* Relative to the untreated native group, MFUT + G + S significantly (*p* < 0.05) outperformed individual treatments and MFUT + G. MFUT + G + S treatment increased PAL (phenylalanine ammonia-lyase), PPO (polyphenol oxidase), POD (peroxidase), and SOD (Superoxide dismutase) activities by 7.07, 9.60, 6.81, and 10.92 folds, respectively, while reduced tannins and phytic acid by 66.37 % and 60.07 %, respectively. These biochemical modifications enhanced polyphenolic accumulation (particularly chlorogenic acid, vanillic acid, syringic acid, rutin, and catechins), antioxidant activity and protein digestibility by 173.98 %, 284.89 %, and 102.21 %, respectively. FTIR and XRD confirmed structural modifications indicated by diminished starch crystallinity index (31.98–13.40 %) and α-helix (10.97–10.25 %) accompanied by enhanced β-sheets (20.71–35.83 %). Furthermore, MFUT + G + S exhibited reduced pasting properties and particle size, alongside increased surface hydrophobicity, zeta-potential and uniform particle dispersity. Collectively, these structural modifications enhanced water holding capacity (295.65 %), water absorption index (30.45 %), swelling power (35.94 %), emulsifying activity (68.04 %) and emulsifying stability (55.30 %) as well as superior thermal and textural stability. Furthermore, E-nose sensor response indicated an improved aroma pattern of MFUT + G + S. Collectively, these findings demonstrate that sequential application of MFUT and soaking pretreatments enhance both the functional quality and digestibility of germinated *Fagopyrum tataricum* flour.

## 1 Introduction

*Fagopyrum tataricum* commonly known as *Fagopyrum tataricum*is extensively cultivated in Bhutan, China, central Europe, Nepal, and northern India due to its higher ecological adaptability, stress resilience, and nutritional importance [1], finding diverse applications in foods and beverage formulations as a natural source of dietary fiber, minerals, and diverse range of bioactive compounds including triterpenoids, d-chiro inositol, bioactive polysaccharides, proteins and peptides are linked with its anti-inflammatory, anti-hypertensive, anti-hyperlipidemic, anticancer, anti-diabetic, angio-protective and hepatoprotective potential [2], [3]. Furthermore, its high-value protein, with a balanced amino acid profile, retains higher levels of essential amino acids, making it a promising ingredient for gluten-free applications [4]. However, the presence of anti-nutritional factors (ANFs), such as tannins, phytic acid, and trypsin inhibitors, limits bioaccessibility of tartary buckwheat’s nutritional and functional components, reducing their bioavailability and imparting bitter flavor to grain [5].

Traditionally, germination have been extensively employed for reducing ANFs in cereals and pseudocereals. Germination improves phenolics and flavonoids via upregulated chalcone isomerase (CHI) and phenylalanine ammonia-lyase (PAL) activity [6] and suppressed rutin-degrading enzymes [7], ultimately leading to improved antioxidant and free radical scavenging activity [8]. Germination for 48–72 h increases polyphenols, phenylalanine, lysine, leucine, and GABA, whereas shorter durations (24–48 h) improve water absorption index (WAI), forming capacity, and glycemic response of buckwheat flour. Ultrasonication, novel physical processing approach, enhances polyphenol levels and modifies grain structure via cavitation which disrupts intercellular matrix to release polyphenols and alters molecular configuration, improving structural and biochemical properties of grains [9]. Whereas, soaking softens the grains through enhanced hydration alongside leaching out ANFs that improves nutrient bioavailability [10].

Soaking and ultrasonication are often used as germination pretreatments, resulting in significant improvements in the nutritional, functional, and structural properties of pseudocereals. Thakur et al.[10] reported that soaking followed germination markedly reduced tannins and phytic acid by 59.91 %,32.30 %,27.08 %, and 17.42 %,29.57 %, 47.57 % in buckwheat, amaranth and quinoa, respectively, along with improved protein and phenolics contents. Another study stated that ultrasound-assisted germination can reduces tannins up to 83 % in buckwheat while boosting its phenolics and antioxidant activity, rendering rutin as the dominant flavonoid, however, extent of reduction is highly dependent on processing conditions [11].

Previous studies have demonstrated that germination [6], soaking [12], and ultrasonication [13] when applied independently, can enhance the nutritional and techno-functional properties of buckwheat flour. Recently, combined bioprocessing strategies have shown improvements in bioactive contents and functionality of cereals and lentils [14], [15], [16]. However, sequential and combined application multifrequency ultrasonication, germination and soaking has not been systematically investigated in *Fagopyrum tataricum*. The existing studies have largely focused on individual or dual effects of these treatments, leaving a gap in understanding how their combined application particularly ultrasonication as pre-germination stimulus followed by soaking influences the metabolic cascade during germination. To address this gap, the present study investigates the individual and combined effect of multifrequency ultrasonication, germination and soaking on polyphenols profile, ANFs, structural characteristics and techno-functional properties of *Fagopyrum tataricum*. We hypothesize that combined treatment will yield superior improvements in functional characteristics compared to individual treatments, thereby enhancing the commercial potential of *Fagopyrum tataricum* for value-added food products.

## 2 Materials and methods

### 2.1 Procurement of raw materials

The seeds of Daliang Shan black *Fagopyrum tataricum* were procured from Leshui, Zhejiang. Healthy seeds were selected; before sample preparation, they were screened to remove foreign particles and rotten seeds.

### 2.2 Sample preparation

#### 2.2.1 Seed disinfection

Healthy *Fagopyrum tataricum* seeds were soaked in sodium hypochlorite (1 %) at 25 °C for 30 min, subsequently, washed with distilled water thrice, and subjected to multifrequency ultrasonication, germination and soaking, the processing condition were optimized based on single-factor optimization Fig. S1.

#### 2.2.2 Multifrequency ultrasonication (MFUT)

The disinfected seeds (50 g) were placed in a polythene zip lock bag with distilled water (150 mL), and ultrasonicated using a multifrequency hexagonal flat-panel divergent ultrasonic bath developed by School of Food and Biological Engineering at Jiangsu University (Zhenjiang, China) using previous method [17]. Briefly, the ultrasonication conditions were as follow: frequency: 20/40/60 KHz, power: 220 W, pulse ratio: 5 s on/off, time: 30 min, temperature: 25 °C, power density: 65 W/L), and frequency mode: sequential.

#### 2.2.3 Germination (G)

The disinfected seeds (50 g) were spread on sterile germination trays lined with moistened muslin cloth and germinated for 72 h in dark germination chamber at with maintained temperature (30 ± 2 °C) and relative humidity (90 ± 5 %). The seeds were rinsed with sterile water (15 mL) every 12 h to retain moisture.

#### 2.2.4 Soaking (S)

The disinfected seeds (50 g) were immersed in distilled water (1:5 (w/v)), in a sterile beaker for 6 h at 25 ± 2 °C in static conditions. Subsequently, the seeds were rinsed and dried with a paper towel to remove excess water.

**Fig. 1:**
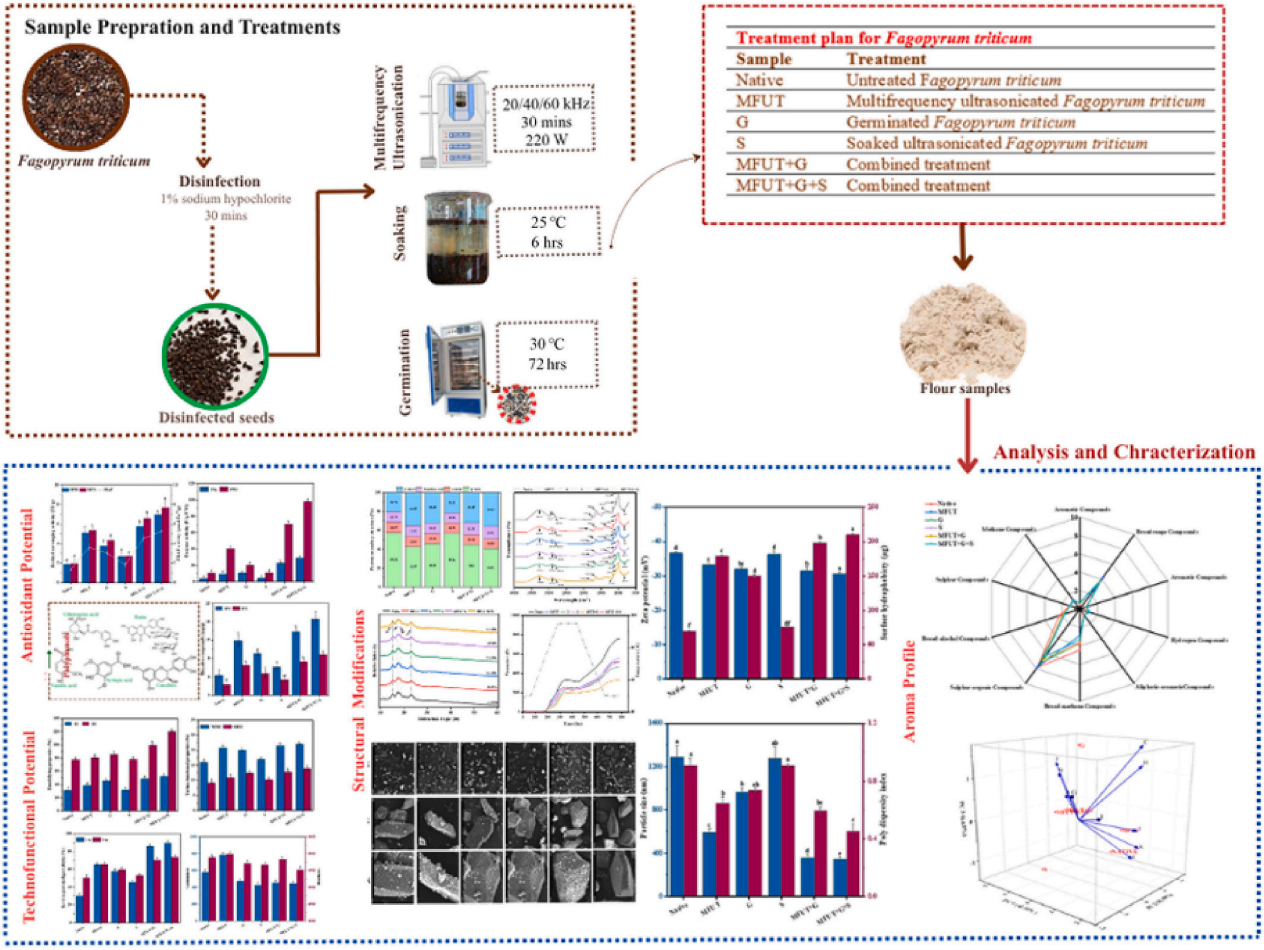
Schematic illustration of the experimental workflow for *Fagopyrum tataricum* (Tartary buckwheat) processing and characterization.

The disinfected seeds, categorized into 6 groups, labelled as, native (untreated *Fagopyrum tataricum*), MFUT, G, S and their combinations (MFUT + G and MFUT + G + S) were freeze-dried at −30 °C, 40 mbar for 48 h using lyophilizer (Scientz-10 N, Ningbo Scientiz biotechnology Co., LTD, China) and a fine powder (120-mesh) was obtained, used for further analysis (Fig. 1).

### 2.3 Phenolics extraction from native and treated Fagopyrum tataricum

The extracts were prepared by extracting sample (1 g) with 20 mL of methanol (80 %) and sonicated (40 kHz, 25 °C, 30 min) followed by centrifugation at 11,180 × g and 4 °C for 10 min. The supernatant was then obtained and stored at −18 °C for phenolics and anti-oxidant analysis [18], [19].

### 2.4 Determination of antioxidant potential of native and treated Fagopyrum tataricum

#### 2.4.1 Total phenolic contents (TPC) and total flavonoid contents (TFC)

TPC and TFC of sample extracts was determined using Folin-Ciocalteu (FC) assay and AlCl_3,_ respectively according to previously established protocol [20]. The absorbance was recorded at 765 nm and 510 nm using a Tecan Spark 10-M multi-mode microplate reader (Tecan Group Ltd., Männedorf, Switzerland) and results were expressed as mg of gallic acid equivalent/mL of sample and mg of rutin equivalent/mL of sample for TPC and TFC, respectively.

#### 2.4.2 DPPH, ABTS, and FRAP radical scavenging activities

The DPPH, ABTS, and FRAP activities of *Fagopyrum tataricum* samples were determined using established protocols [17]. For the DPPH assay, 240 µL of the sample was homogenized with 5 mL of the DPPH solution (0.1 mM), rested for 25 min at 25 °C, and the absorbance was recorded at 517 nm. For the FRAP assay, 0.6 mL of FRAP solution (0.3 M acetate buffer, 10 mM TPTZ, 20 mM FeCl_3_·6H2O) was added to 2 mL of the sample and 0.6 mL of H2O, and the mixture was incubated at 37 °C for 30 min. Afterwards, the absorbance was taken at 595 nm. For the ABTS assay, 20 µL of the sample was mixed with 200 µL of the ABTS working solution, incubated in the dark for 5 min, and the absorbance was measured at 734 nm. The results were expressed as µg Trolox equivalents (TE)/g for DPPH and ABTS, whereas µmol Fe^2^⁺/g for FRAP.

### 2.5 Identification & quantification of free phenolics of native and treated Fagopyrum tataricum

The identification of individual phenolic compounds was performed using an HPLC-UV Agilent 1260 Infinity II system. The extracts were prepared in section 2.3. were eluted through a Zorbax C-18 column (4.6 mm: 250 mm: 5 mm). The mobile phase mixture consisted of Mobile phase A (0.1 % acetic acid) and Mobile phase B (100 % acetonitrile). The linear gradient protocol was set as follows: 0–10 min, 5–10 % solvent B; 10–15 min, 10–20 % solvent B; 15–25 min, 20–38 % solvent B; 25–30 min, 38–40 % solvent B; 30–31 min, 40–100 % solvent B; 31–35 min, 100 % solvent B; 35–36 min, 100–5 % solvent B; 36–50 min, 5 % solvent B. The flow rate was 0.8 mL/min, whereas the column temperature was maintained at 30 ℃. The HPLC chromatograms were recorded at 284 nm for flavonoids and at 250 nm for phenolics. The phenolic compounds were identified by comparing retention times and UV–VIS spectra with calibration curves of cinnamic acid, syringic acid, vanillic acid, coumaric acid, ferulic acid, chlorogenic acid, gallic acid, rutin, catechins and quercetin standards, showing linearity with R^2^ = 0.99, 0.99, 0.98, 0.99, 0.99, 0.99, 0.99, 0.99, 0.99, and 0.99, respectively [21].

### 2.6 Determination of antinutrients in native and treated Fagopyrum tataricum

#### 2.6.1 Determination of tannin contents

Briefly, 0.2 g sample was extracted with 6 mL water (100 °C, 30 min), centrifuged (2000 × *g*, 5 min). The supernatant was diluted (1:1), 0.2 mL of this was mixed with 1 mL water, 0.2 mL Folin-Denis (FD) reagent and 0.6 mL of Na_2_CO_3_, rested for 40 min at 4 °C and absorbance was recorded at 765 nm [22].

#### 2.6.2 Determination of phytic acid contents

Briefly, 0.35 g sample was extracted with 7 mL of 2.4 % HCl (210 rpm, 130 min) and centrifuged (1000 × *g,* 20 min, 10 °C). The supernatant was mixed with 0.7 g NaCl (210 rpm, 35 min), incubated at −20 °C for 20 min and centrifuged again under same conditions. The supernatant was diluted with 24 mL of water; 5 mL of this solution was mixed with 4 mL Wade reagent, incubated for 20 min at room temperature, and absorbance was recorded at 500 nm [23].

### 2.7 In-vitro protein digestibility of native and treated Fagopyrum tataricum

Protein digestibility was determined using slightly modified method of Zhang et al. [24]. Briefly, 100 mg sample was extracted with HCl (0.1 M, 10 mL) and pepsin (1.5 mg) at 37 °C, after1 hr 1 mL aliquot was taken and heat-inactivated. pH of remaining mixture was adjusted to 8 with NaOH (0.2 M) and mixed with pancreatin (5 mg) and phosphate buffer (7 mL), incubated at 37 °C, after 2 hr another 1 mL aliquot was taken and heat-inactivated. Subsequently, the aliquots were centrifuged (8000 × g, 10 min, 4 °C) and degree of hydrolysis was determined through OPA assay at 340 nm using serine as standard.

### 2.8 Techno-functional properties of native and treated Fagopyrum tataricum

Water and oil holding capacity was determined by centrifuging (3000 *× g*, 25 min) the water and oil homogenate (1 g sample and 10 mL water/oil), and calculated as water or oil retained per g dry flour. Water absorption index (WAI), water solubility index (WSI), and swelling properties (SP) were determined by heating water homogenate (95 °C, 15 min) and centrifuging (3000 × *g,* 10 min), supernatant was dispensed onto a pre-weighed evaporating aluminum dish for 12 h at 110 °C to determine soluble solid content [25]. WAI, WSI, and SP were calculated using Eqs. (1)–(3), respectively.

(1) $$\mathrm{WAI}=\frac{\mathrm{Wss}}{\mathrm{Wo}}$$

(2) $$\mathrm{WSI}=\frac{\mathrm{Wds}}{\mathrm{Wo}}\times 10$$

(3) $$\mathrm{SP}=\frac{\mathrm{Ws}}{\mathrm{Wo}-Wds}$$

where:

Wo: initial sample weight (g); Wss: weight of supernatant + aluminum dish (g); Wds: final weight of dried sample (g); Ws: weight of sediment (g).

For emulsifying activity (EA) and emulsion stability (ES), sample (0.7 g) was mixed with 10 mL of distilled water and 10 mL of corn oil, homogenized (1000 rpm, 1 min) using an Ultra-Turrax T25 homogenizer (IKA, Staufen, Germany), and centrifuged (1300 × *g*, 5 mins) EA was calculated using Eq. (4). Tubes were heated at 80 °C for 30 min and kept for 30 min, then centrifuged under same conditions, and ES was calculated using Eq. (5) [26].

(4) $$\mathrm{EA}\;\left(\%\right)=\frac{\mathrm{emulsion}\;volume}{\mathrm{Total}\;intial\;volume}\times 100$$

(5) $$\mathrm{ES}\;\left(\%\right)=\frac{\mathrm{emulsion}\;volume\;after\;heating}{\mathrm{Total}\;intial\;volume}\times 100$$

### 2.9 Zeta potential, particle size, polydispersity index, and surface hydrophobicity of native and treated Fagopyrum tataricum

Zeta potential, particle size, polydispersity index of diluted samples (1:100) were analyzed using Anton Paar Litesizer 500 particle size analyzer as previously reported [27]. Surface hydrophobicity was determined as bromophenol blue binding capacity (μg) using method of Wang et al. [28]and calculated as:

Surfacehydrophobicity(μg)=200(Ablank-Asample)/Ablank

### 2.10 Physical properties of native and treated Fagopyrum tataricum

#### 2.10.1 Color profile analysis

Digital colorimeter (SC–80C, Beijing Kangguang Optical Instrument Co, Ltd) was used to measure color coordinates of samples and articulated as L* (lightness), a* (redness), and b* (yellowness), and overall color difference (ΔE), calculated as per following equation:

ΔE = [(L*- L*_ref_)^2^ + (a*- a*_ref_)^2^ + (b*- b*ref)^2^]^1/2^

#### 2.10.2 Texture profile analysis

Briefly, 10 g sample was kneaded into circular dough balls using 10 mL of distilled water. The texture parameters hardness, adhesiveness, resilience, cohesion, springiness, gumminess, and chewiness of the resultant dough were analyzed using a texture analyzer (TA.XT Plus, Stable Micro Systems, UK) as previously established protocol [29], with a few modifications. The S/50S aluminum probe was utilized under the following conditions: applied force strain (5 g), strain (40 %), pre-test speed (2.00 mm · s⁻^1^), post-test speed (1.00 mm·s⁻^1^), and 2 force cycles with a resting gap of 2 s.

### 2.11 Structural properties of native and treated Fagopyrum tataricum

#### 2.11.1 Fourier transform infrared spectroscopy (FTIR)

The *Fagopyrum tataricum* samples were positioned on a console equipped with a germanium crystal ATR-FTIR spectrometer (Thermo Nicolet Co., Nicolet iS50, USA). Before sample analysis, the instrument was calibrated for wavenumber accuracy. Afterwards, spectra were recorded over 32 scan runs at 4 cm^−1^ spectral resolution, spanning wavelengths from 4000 to 500 cm^−1^. Background scans of clean ATR crystal were recorded and automatically subtracted for each sample. Spectral analysis and processing were performed using OMNIC 9.0 and Peak Fit software (Thermo Fisher Scientific, Waltham, MA, USA) [30].

#### 2.11.2 X-ray diffraction analysis (XRD)

The samples were placed on scanning discs and subjected to an X-ray diffractometer (Ultima IV, Rigaku, Japan). The X-ray tube was operated at 40 kV and 40 mA, with a scanning range of 10–60° at a scan speed of 5°/min.

#### 2.11.3 Scanning electron microscopy (SEM)

The gold-plated native and treated *Fagopyrum tataricum* samples were scanned using an SEM (SU8000TMP, Hitachi, Japan) at an acceleration voltage of 5 kV, and micrographs were taken at magnifications of 100X, 500X, and 1000X.

### 2.12 Thermal properties (DSC) of native and treated Fagopyrum tataricum

5 mg of each sample was mixed with 10 µL of water in an aluminum pan and left to rest overnight. A DSC scan was performed from 30 to 100 °C at 5 °C min^−1^. Each sample was scanned twice, and thermal properties were recorded using an empty pan as a control [31].

### 2.13 Pasting properties of native and treated Fagopyrum tataricum

Pasting properties were evaluated using a rapid viscosity analyzer according to previously established protocol [22]. Sample (4 g) was weighed into RVA aluminum canister containing 26.35 mL distilled water. The mixture was homogenized and subsequently loaded into RVA for analysis using a standard heating–cooling profile.

### 2.14 Aromatic profile of native and treated Fagopyrum tataricum using E-nose

The E-nose system was used to analyze the aromatic profile of *Fagopyrum tataricum* samples, constituting a multi-sensor array, W1C, W5C, W3C, W6S, W5C, W1S, W1W, W2S, W2W, and W3S, detecting aromatic, broad-range, aromatic, hydrogen, aliphatic- aromatic, broad methane, sulphur-organic, broad-alcohol, sulphur-chlorine, and methane-aliphatic compounds, respectively, with control serving as a baseline. Prior to each experiment, sensors were flushed with dry air for 60 s, stabilized for 30 sed to establish base line, and purged with dry air for 120 s between samples to prevent carryover. Principal Component Analysis (PCA) was used to reduce data dimensionality, followed by cluster analysis to classify samples based on sensor responses [21].

### 2.15 Statistical analysis

The study followed an independent biological replication design; each treatment was applied to three separately processed batches, and results were presented as mean ± standard deviation (n = 3). Statistical Package OriginPro 8.5 was used to determine the significance level (*p* < 0.05) and to implement one-way ANOVA to assess variance, followed by Tukey’s Test for mean comparisons.

## 3 Results and discussion

### 3.1 Antioxidant potential of native and treated *Fagopyrum tataricum*

#### 3.1.1 TPC and TFC of *Fagopyrum tataricum*

**Fig. 2:**
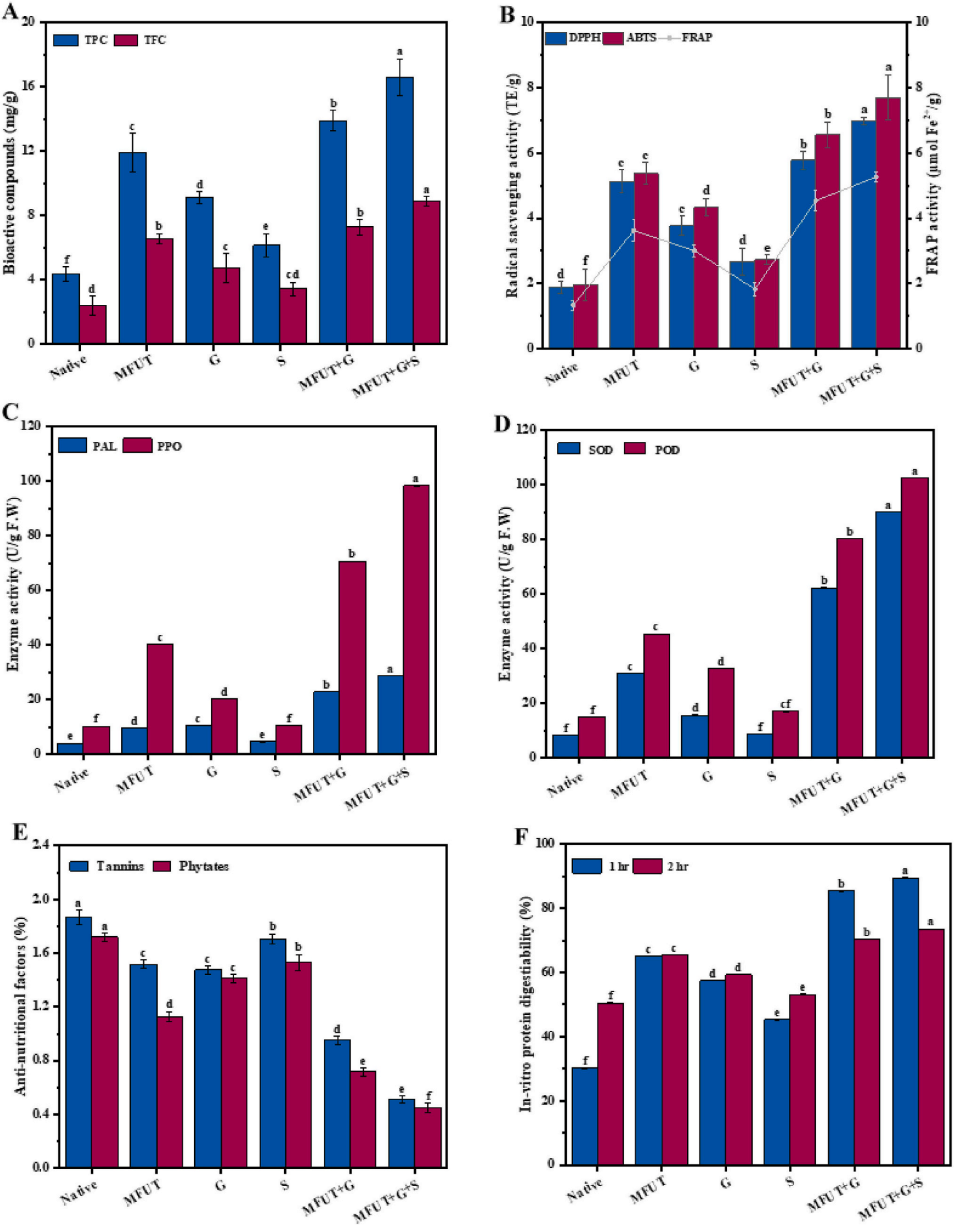
The bioactive compounds, antioxidant potential, enzyme activities, anti-nutritional factors and in-vitro protein digestibility of tartary buckwheat subjected to ultrasonication, germination, soaking and combined treatments. A. TPC and TFC contents of treated tartary buckwheat. B. DPPH, ABTS, and FRAP activities of treated tartary buckwheat. C. PAL and PPO activity of treated tartary buckwheat. D. SOD and POD activity of treated tartary buckwheat. E. Tannins and phytates of treated tartary buckwheat. F. in-vitro protein digestibility of treated tartary buckwheat. Native: untreated tartary buckwheat, MFUT: multifrequency ultrasonication, G: germination, S: soaking. Lowercase letters represent a significant difference (*p* < 0.05; Tukey’s test).

Polyphenols are secondary metabolites widely recognized for their potent antioxidant capacity, primarily associated with metal chelation and free radical scavenging, with phenolic acids and flavonoids are principal bioactive subclasses. The combined treatment (MFUT + G + S) significantly (*p* < 0.05) enhanced both TPC (4.37–16.61 mg GAE/g) and TFC (2.41 to 8.88 mg of QE/g), corresponding to an increase of 280 % and 269 %, respectively, compared to untreated *Fagopyrum tataricum* Fig. 2A. This enhancement can be attributed to cavitational shear stress, disrupting cell walls, facilitating the release of bound bioactive compounds, thereby increasing phenolic contents [32], [33]. Furthermore, germination upregulates phenylpropanoid pathway by activating phenylalanine ammonia‑lyase (PAL) promoting the synthesis of phenolics and flavonoids [34], corroborated with our results that highest PAL activity (Fig. 2C) in MFUT + G + S and MFUT + G is associated with enhanced bioactive compounds in combined treatments compared to native group. Supporting this, Tufail et al. [17] demonstrated thatultrasound-assisted germination upregulates expression the expression of key phenylpropanoid pathway genes including *mPAL*, *mC3H*, *mCHS*, and *mC4H* in barley fractions, thereby enhancing phenolics biosynthesis. Furthermore, ultrasonication-generated free radicals may act as signaling molecules that stimulate PAL activity, thereby priming the phenylpropanoid pathway for enhanced phenolics production [35]. Comparable trends have been reported in ultrasound-assisted germination studies on oats seeds and radish seeds [22], [36], affirming that observed elevation in phenolic and flavonoids levels is a consistent response across various plants species subjected to combined treatment. The enhanced TPC and TFC in MFUT + G + S (16.61 mg GAE/g and 8.88 mg of QE/g) compared to MFUT + G (13.89 mg GAE/g and 7.27 mg of QE/g), may be attributed to post-ultrasound soaking phase, which promotes efficient tissue hydration and reactivation of hydrolytic and phenylpropanoid enzymes, thereby augmenting phenolic biosynthesis during subsequent germination [37].

#### 3.1.2 DPPH, ABTS, and FRAP radical scavenging activities of *Fagopyrum tataricum*

Antioxidant capacity denotes the intrinsic potential of bioactive compounds to quench free radicals and alleviate oxidative stress, preserving cellular integrity. The antioxidant activity was assessed using DPPH, ABTS, and, FRAP radical scavenging assays, showing a significant increase across all treatment groups relative to native group as depicted in Fig. 2B. The combined treatments MFUT + G + S and MFUT + G exhibited an increase of 272.43 %, 289.85 %, and 295.08 % and 207.43 %, 232.30 %, and 240.13 % for DPPH, ABTS, and FRAP activities, respectively compared to native group. The antioxidant efficacy of polyphenols is primarily attributed to their ability to donate H-atoms to reactive species, thereby stabilizing free radicals, governed degree of hydroxylation, wherein hydroxyl group abundance on aromatic ring enhances electron donation [38]. In agreement with structure–function relationship, the elevated TPC and TFC in combined treatment groups corresponded with superior radical scavenging activity.

The observed positive association between phenolic availability and antioxidant capacity is further reinforced by congruent evidence from ultrasound-assisted germinated cereals and legumes [32], [39], [40]. This broad concordance across diverse plant matrices lends considerable weight to the propose structure–function activity and supports that germination and ultrasound-based strategies are broadly effective for enhancing bioactive potential of cereals.

### 3.2 Antioxidant enzyme activities of native and treated *Fagopyrum tataricum*

Superoxide dismutase (SOD), peroxidase (POD), and polyphenol peroxidase (PPO) are key enzymatic biomarkers of oxidative stress and constitutes the primary antioxidant defense system in plants, where oxidative conditions trigger both enzymatic and non– enzymatic antioxidant responses, thereby enhancing antioxidant enzyme activities [41]. The antioxidant enzyme activities were significantly enhanced across all treatments, with combined treatments exhibiting the most pronounced increase (Fig. 2C and D). PAL activity increased from 4.05 to 28.65 U/g in MFUT + G + S treatment, indicating 7-folds enhancement. This augmentation can be attributed to ultrasound induced oxidative stress, which triggers plant defense mechanism and upregulates phenylpropanoid metabolism [42]. Qiao et al. [43] demonstrated that ultrasound causes water molecule splitting, generative ROS, inducing oxidative damage, consequently enhancing the generation of secondary metabolites by regulating phenylpropanoid pathway genes. Similarly, [44] reported that ultrasound assisted germination significantly increased TPC and antioxidant activity in buckwheat by accelerating enzyme activities and biochemical changes through cellular modifications. POD and PPO activities followed a similar pattern with MFUT + G + S achieving highest values 102.56 and 98.36 U/g, respectively compared to control (15.05 and 10.25 U/g). MFUT + G + S exceeded MFUT + G by 28 5 for POD and 39 % for PPO. This enhancement possibly to role of PAL catalyzing conversion of L-phenylalanine to *trans*-cinnamic acid, precursor of various phenolic compounds, while PPO oxidizes phenols to quinones contributing to plant stress resilience [45], [46]. Hossain et al. [47] observed comparable increase in antioxidant enzyme activities in *Fagopyrum tataricum*under drought, with PAL and PPO levels increasing by 164 % and 154 %, respectively. SOD, primary enzymatic oxidant responsible for catalyzing superoxide radicals to H_2_O_2_, increased from 8.25 U/g (native) to 90.15 U/g in MFUT + G + S. These 10-folds increase reflects plant activated defense response to cavitation induced oxidative stress. The moderate increase observed in individual treatments confirms that each treatment stimulates antioxidant, enzymes activities their sequential application produces pronounced effects (Fig. 2C and D). The hierarchical response across all four enzymes demonstrates that ultrasound acts as an effective abiotic elicitor, while germination provides enzymatic mobilization and soaking facilitates hydration leading to coordinated activation of phenylpropanoid pathway and antioxidant defense system [17].

### 3.3 Individual phenolic profile of native and treated *Fagopyrum tataricum*

Polyphenols, naturally occurring plant metabolites, are associated with a diverse range of therapeutic effects. HPLC-UV was employed to analyze the phenolic metabolites in native and treated TB samples, presented in Table 1. The combined treatment MFUT + G + S (7234.19 µg/g) and MFUT + G (µg/g) has shown a significant (*p* < 0.05) increase in total polyphenols compared to native (2640.33 µg/g). The observed polyphenol enhancement aligns with established effects of combined bioprocessing, wherein ultrasonication disrupts cellular structure, release bound phenolics and triggers enzymatic defense, while germination promotes synthesis and mobilization of these phenolics [48]. Our findings further corroborate this mechanism, demonstrating not only quantitative increase in TPC but also qualitative shift in phenolic profile, notably, a marked elevation in chlorogenic acid (6.42–7.71 µg/g), vanillic acid (10.57–12.11 µg/g), syringic acid (4.52–15.55 µg/g), rutin (2250.62–6789.81 µg/g), and catechins (38.05–62.52 µg/g) indicates that combined treatments effectively promote bioconversion of these phenolics into bioavailable and antioxidant active forms. The enhanced radical scavenging activity observed in combined treatments (Fig. 2B) can be attributed not only to increased TPC but also to specific compositional enrichment. Among the identified compounds, catechins and chlorogenic acid emerged as key contributors. Whereas, the *ortho*-dihydroxyl groups on catechin B-ring facilitates electron donation [49], while the extended conjugated system of chlorogenic acid stabilizes the resultant phenoxyl radicals, amplifying its antioxidant efficacy [50]. Similarly, rutin augments radical quenching through its catechol-bearing quercetin aglycone, a potent electron donor; concurrently, its rutinose disaccharide moiety enhances solubility and binding affinity to macromolecules, thereby improving techno-functional properties [51]. In contrast, syringic acid, a di-methoxy derivative of gallic acid exhibits distinct structural feature, wherein a hydroxyl group is flanked by two methoxy substituent. This unique O-methylation pattern not only confers enhanced radical stability but also modulates precise binding interactions, manifesting its diverse pharmacological potential [52]. Collectively, these structure-dependent activities corroborate the observed positive correlation between phenolic enrichment and functional antioxidant performance.

**Table 1:** Effect of multi-frequency ultrasonication, soaking, germination and their combined treatments on phenolic acids and flavonoids contents of tartary buckwheat.

Phenolic compounds	RT (min)	Native	MFUT	G	S	MFUT + G	MFUT + G + S
**Phenolic acids (µg/g)**							
Cinnamic acid	27.2	7.14 ± 0.06^b^	7.01 ± 0.07^c^	6.52 ± 0.08^c^	6.54 ± 0.06^c^	7.04 ± 0.06^b^	8.66 ± 0.08^a^
Syringic acid	17.2	4.52 ± 0.08^e^	10.46 ± 0.12^d^	13.31 ± 0.17^c^	10.50 ± 0.13^d^	15.98 ± 0.18^a^	15.55 ± 0.15^b^
Vanillic acid	16.2	10.57 ± 0.13^d^	14.82 ± 0.18^b^	16.07 ± 0.18^a^	15.09 ± 0.15^b^	16.36 ± 0.15^a^	13.30 ± 0.15^c^
Coumaric acid	20.2	12.14 ± 0.08^c^	11.72 ± 0.13^d^	13.08 ± 0.13^a^	12.57 ± 0.13^b^	12.59 ± 0.13^b^	12.11 ± 0.13^c^
Ferulic acid	21.5	291.9 ± 1.36^e^	393.67 ± 1.76^b^	393.67 ± 1.76^b^	380.82 ± 1.91^c^	567.17 ± 1.88^a^	303.08 ± 1.65^d^
Chlorogenic acid	14.87	6.42 ± 0.11^e^	8.03 ± 0.13^c^	7.63 ± 0.13^d^	8.39 ± 0.13^b^	9.18 ± 0.13^a^	7.71 ± 0.13^cd^
Gallic acid	3.11	16.04 ± 0.31^d^	20.16 ± 0.33^a^	20.43 ± 0.33^a^	14.77 ± 0.25^b^	18.13 ± 0.33^b^	19.08 ± 0.33^e^

**Flavonoids (µg/g)**							
Rutin	21.4	2250.62 ± 572^f^	2859.36 ± 6.47^d^	3400.71 ± 7.85^c^	2531.17 ± 6.40^e^	4021.96 ± 7.93^b^	6789.81 ± 9.89^a^
Catechins	15.8	38.05 ± 0.25^f^	43.78 ± 0.33^e^	55.02 ± 0.33^b^	47.98 ± 0.33^d^	51.14 ± 0.3^c^	62.52 ± 0.38^a^
Quercetin	27.6	2.86 ± 0.04^b^	3.13 ± 0.04^a^	2.54 ± 0.04^c^	2.31 ± 0.04^e^	2.63 ± 0.04^d^	2.37 ± 0.04^e^
**Total Polyphenols**	−	2640.33	3372.13	3928.97	3030.14	4722.17	7234.19

Native: Raw buckwheat; MUFT: multi-frequency ultrasound treatment; G: germination, S: soaking; RT: Retention time.

### 3.4 Anti-nutritional factors and in-vitro protein digestibility of native and treated *Fagopyrum tataricum*

Phytic acid and tannins represent the primary anti-nutritional factors in cereals and pseudocereals, characterized by their ability to form indigestible complexes with protein and minerals, thereby compromising nutrient bioavailability [53]. Compared to native group all treatments showed significant reductions in both tannins and phytic acid contents, as presented in Fig. 2E. Combined treatments, MFUT + G + S significantly (*p* < 0.05) reduced tannins contents from 1.87 to 0.51 % and phytic acid content from 1.72 to 0.44 % (on dry weight basis), indicating reduction of 72.67 % and 73.82 % relative to native group. Whereas, corresponding reduction of 65.46 % and 68.15 % in tannins and 66.37 % and 60.07 % in phytic acid compared to individual germination and MFUT groups, respectively. The superior effect observed in MFUT + G + S can be attributed to ultrasound-induced cavitation, enzymatic degradation and efficient solute leaching. Mechanistically, acoustic cavitation generates localized shear forces and microstreaming disrupting cellular matrices, facilitating leaching of soluble ANFs. Concurrently, germination activities endogenous enzymes phytase [54] and polyphenol oxidases (Fig. 2D), hydrolyzing phytate complexes and oxidize phenolics based ANFs, respectively. Furthermore, post-ultrasound soaking optimizes enzymatic activity and leaching as presented in (Supplementary Table S1). Our findings align with established trends in cereals and legumes, wherein combined bioprocessing approaches have been shown to cause substantial reduction in ANFs (80–90 %), although the magnitude of reduction remains contingent upon processing parameters including germination duration, ultrasonication power and time, and genotype specific compositional factors [11], [55].

Considering that tannins and phytic acid interfere with nutrients bioavailability and digestibility by forming indigestible complexes, the functional implications of ANFs reduction were assessed by evaluating the *in-vitro* protein digestibility (IVPD) of treatment groups (Fig. 2F). Initial phase of digestion the combined treatments MFUT + G + S and MFUT + G exhibited the highest IVPD of 89.45 % and 85.35 % compared to native group (30.15 %). This trend aligns with established negative correlation between ANFs content and protein digestibility, wherein ANFs form indigestible complexes with proteins and proteolytic enzymes, limiting substrate accessibility (He). In later digestion phase, MFUT + G + S (73.56 %) and MFUT + G (70.25 %) retained higher IVPD than other treatments, though a modest decline was observed relative to earlier values, attributed to extensive hydrolysis achieved during the initial phase, which depleted accessible substrate and may have led to product-mediated suppression of further enzymatic activity. The superior IVPD in combined treatments can be attributed to cavitational forces disrupting protein–polyphenol aggregates, enhancing protease accessibility to peptide bonds [56]. Simultaneously, germination activates endogenous phytase and protease, hydrolyzing phytate-mineral complexes and storage proteins into more digestible forms [57], further amplified by post ultrasonication soaking, leaching ANFs and sustained enzymatic activity. The IVPD values obtained are comparable to those reported for ultrasonicated-germinated little millet (>82 %) [58] and are consistent with enhanced digestibility observed in germinated amaranth (83.58 %) [59]. Notably, the incremental improvement of MFUT + G + S over MFUT + G (4.83 %) suggests that soaking between ultrasonication and germination plays a critical role in maximizing enzymatic potential, likely by facilitating through hydration and reactivation of metabolic processes [37].

### 3.5 Techno-functional properties of native and treated *Fagopyrum tataricum*

**Fig. 3:**
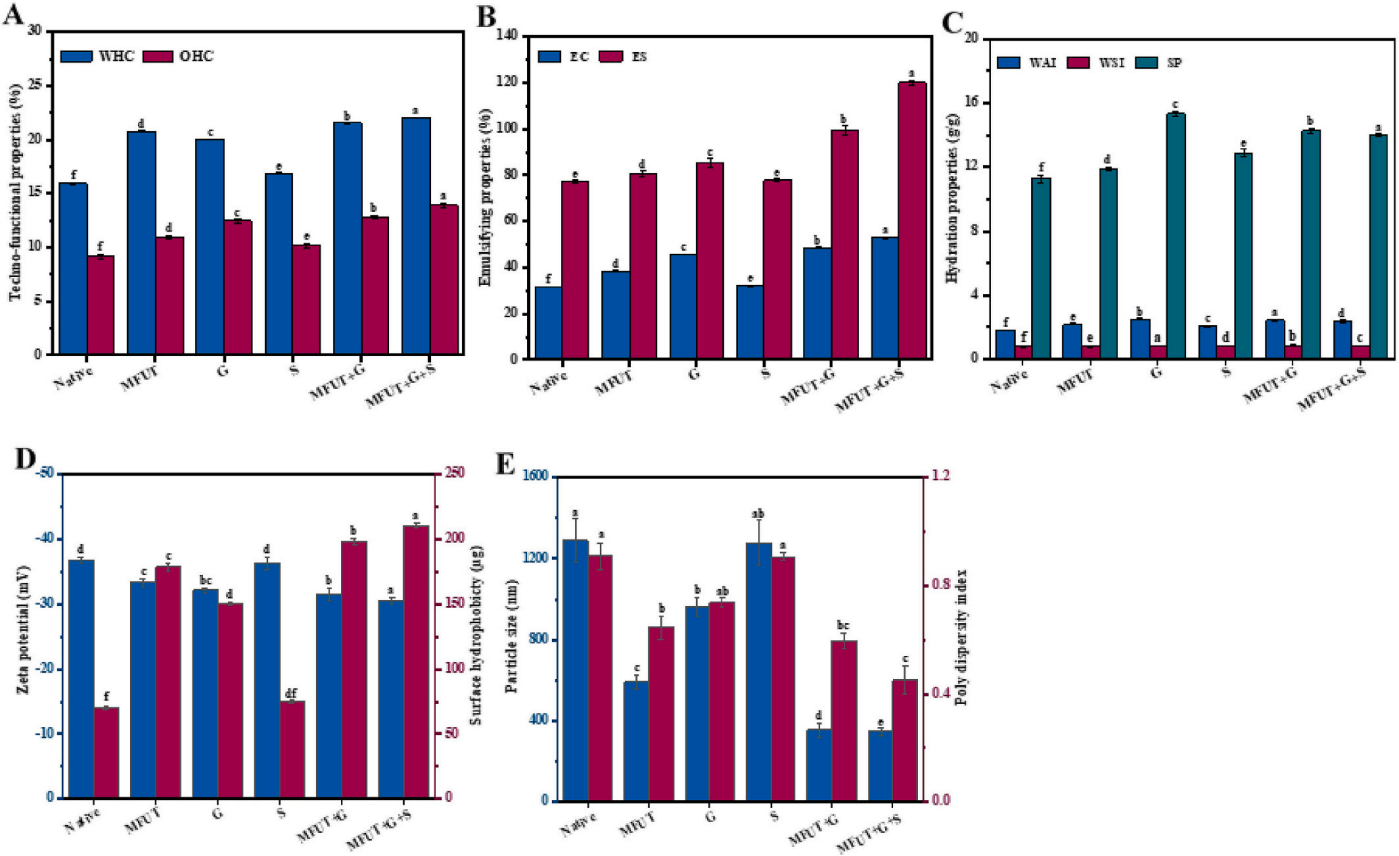
Techno-functional and physiochemical properties of tartary buckwheat subjected to ultrasonication, germination, soaking and combined treatments. A. WHC and OHC of treated tartary buckwheat. B. EC and ES of treated tartary buckwheat. C. WAI, SP, and WSI of treated tartary buckwheat. D. Zeta potential and surface hydrophobicity of treated tartary buckwheat. E. particle size and polydispersity index of treated tartary buckwheat. Native: untreated tartary buckwheat, MFUT: multifrequency ultrasonication, G: germination, S: soaking. Lowercase letters represent a significant difference (*p* < 0.05; Tukey’s test).

The hydration properties including WHC, WAI, WSI and SP are essential indicators of hydration behavior of cereal flours, reflecting their ability to absorb water and release soluble components, essential for functional food formulations [60]. As presented in Fig. 3A and C combined treatment MFUT + G + S exhibited significant improvements in hydration properties, indicating an increase of 295.65 %, 30.45 %, 3.53 %, and 35.94 % in WHC, WAI, WSI, and SP, respectively. Ultrasonication improves hydration properties through disruption of crystalline starch regions, yielding less compact granular arrangements and reduced particle size, along with partially denatured proteins increasing the surface area available for water interactions [61]. While, the germination facilitates enzymatic degradation of protein and starch networks exposing buried hydrophilic sites, thereby enhancing their water-binding capacity [62]. Previous studies have also reported similar trend for millet and sorghum under combined treatments [63], [64].

The oil-holding capacity (OHC) of flour is critical to its functional characteristics, determining its ability to retain oil and, consequently, influencing the products' mouthfeel and texture. MFUT + G + S and MFUT + G treatments achieved 13.92 % and 12.81 % OHC, relative to native group, indicating an increase of 52.01 % and 39.92 %, respectively. The enhancement is likely attributed to high surface area and elevated physical sites for oil entrapment, corroborated by our experimental results for reduced particle size (Fig. 3E) and increased surface hydrophobicity (Fig. 3D), consistent with previous findings on plant matrices [65], [66].

**Fig. 4:**
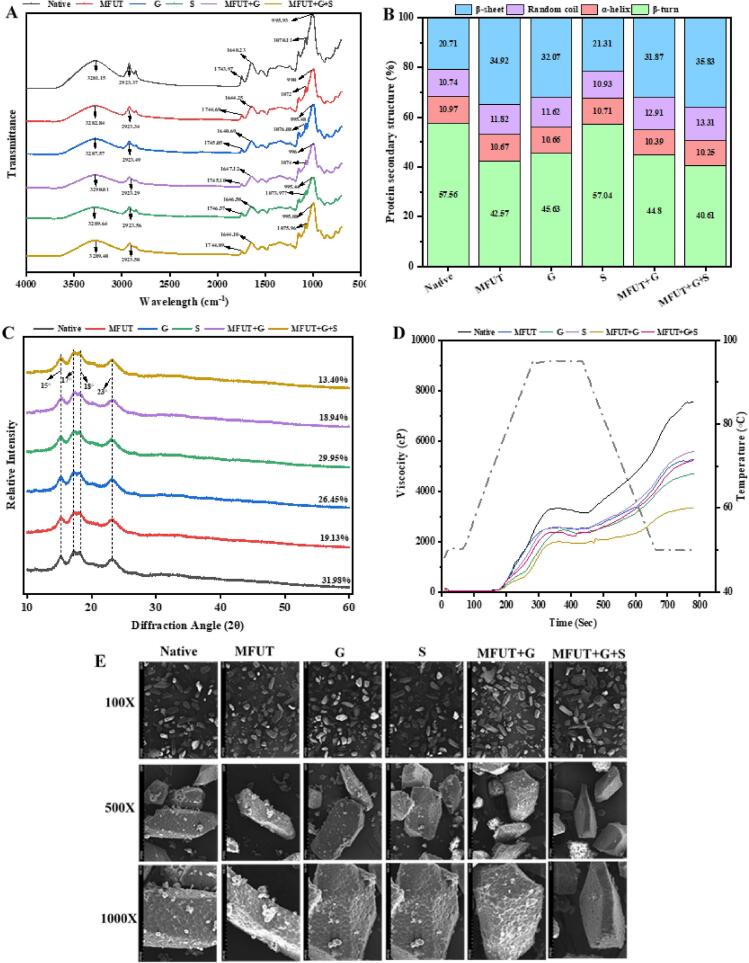
The structural and pasting characteristics of tartary buckwheat subjected to ultrasonication, germination, soaking and combined treatments. A. FTIR spectra of treated tartary buckwheat. B. Protein secondary structure of treated tartary buckwheat. C. X-ray diffraction analysis and crystallinity index of treated tartary buckwheat. D. pasting properties of treated tartary buckwheat. E. SEM micrograms of treated tartary buckwheat. Native: untreated tartary buckwheat, MFUT: multifrequency ultrasonication, G: germination, S: soaking.

The formation of stable emulsion system is essential for multiphase food systems. Emulsifying capacity (EC) reflects protein's adsorption at oil–water interface, while emulsion stability (ES) indicates resistance to phase separation. The combined treatments MFUT + G + S and MFUT + G exhibited an increase of 55.30 % and 68.03 % and 54.55 % and 28.65 % for EC and ES, respectively relative to native group (Fig. 3B). This improvement in emulsifying properties is attributed to protein unfolding, evidenced by decreased α-helix and increased β-sheet (Fig. 4B) along with starch disruption (Fig. 4C) [67], [68]. These conformational modifications enable exposed hydrophobic moieties (Fig. 3D) to migrate to surface of smaller soluble particles, allowing better oil–water interface stabilization [69]. Beyond molecular modifications, the enhanced emulsifying properties of combined treatments may also be ascribed to their higher polyphenols, bearing multiple hydroxyl groups, can interact with proteins via hydrophobic associations, modulating molecular flexibility and reinforcing the emulsion system through enhanced adsorption and steric [70].

### 3.6 Zeta potential, particle size, polydispersity index, and surface hydrophobicity of native and treated *Fagopyrum tataricum*

The zeta potential of all treatment groups remained negative, indicating stable colloidal dispersions with net negative surface charges (Fig. 3D). Native value exhibited the highest absolute zeta potential (−36.73 mV), with progressive reduction across individual treatments with MFUT + G + S displayed the lowest value (−30.64 mV). The moderate reduction can be attributed to cavitation induced structural disruption, exposing positively charged group within protein interior, thereby neutralizing net negative charge [71], whereas, germination induced proteolysis also modifies surface charge distribution [72]. Despite observed reduction, all treated samples-maintained zeta potential exceeding −25 mV, suggesting sufficient electrostatic repulsion for maintaining dispersion stability [73].

Particle size significantly (*p* < 0.05) decreased across all treatments, with native flour exhibiting the largest particle size (1289.88 nm) and MFUT + G + S showing the smallest particle size (347.01 nm), attributed to shear forces disrupting protein aggregates and larger flour particles into smaller fragments [74]. Germination alone significantly contributed to reduced particle size (1289.88–961.55 nm), consistent with findings of Wang et al. [72], who demonstrated decreased particle size in germinated black bean proteins due to enzymatic breakdown of storage proteins. The corresponding reduction in polydispersity index from 0.91 (native) to 0.45 (MFUT + G + S) indicates a more homogenous particle size distribution in combined treatments. The reduction in particle size and PDI, alongside adequate zeta potential favorably contributes to enhanced emulsion capacity and stability observed in treated samples (Fig. 3D and E). Smaller particles promote rapid interfacial adsorption and denser film formation, while uniform distribution reduce coalescence, and moderate negative charge prevents aggregation, enhancing emulsion stability.

Surface hydrophobicity (H_0_) serves as a critical indicator of protein tertiary structure and exposure of non-polar ammino acid residues, was computed as bromophenol blue binding capacity. As shown in Fig. 3D, native group exhibited a baseline H_0_ value of 70.02 µg. MFUT and G significantly increased H_0_ to 178.58 µg and 150.46 µg, respectively. Whereas, combined treatments outperformed individual treatments achieving highest values of 198.41 µg and 210.35 µg, indicating an increase of 183.36 % and 200.41 %, respectively relative to native group. The substantial increase in H_0_ is attributed to protein unfolding as shown in (Fig. 4B), thereby exposing previously buried hydrophobic regions to surface for interactions. These structural rearrangements are consistent with Lu et al. [75] who demonstrated that ultrasound increased surface hydrophobicity and β-sheets while reduced α-helix in lotus seeds protein and Hu et al. [76], who reported enhanced surface hydrophobicity in germinated sesame seeds due to enhanced hydrophobic residues.

### 3.7 Physical characteristics of native and treated *Fagopyrum tataricum*

#### 3.7.1 Color characteristics of native and treated *Fagopyrum tataricum*

The effect of multifrequency ultrasonication, soaking, germination and their combined treatments on color profile (L*, a*, b*) of TB is presented in Table 2. Compared to native group, significant (*p* < 0.05) variations were observed in L*, a*, and b*, possibly associated with pigment degradation or oxidative reactions. The L* value (lightness) decreased across all the treatments (73.72–68.47), is likely attributable enzymatic browning reactions particularly polyphenol oxidase mediated oxidation of phenolics to quinones. Comparable trends have been documented in germinated red beans and ultrasonicated rice [77], [78]. The a* (red-green axis) decreased across all treatments except for MFUT, which exhibited a slight increase from 1.14 to 1.31. This trend is consistent with germination induced changes observed in grass-pea flour [24] and red rice [79], where a* values decreased during sprouting. The increase in a* for individual MFUT treatment, may attributed to phenolics leaching and their subsequent oxidation into brown pigments by activated polyphenol oxidase [80]. In contrast, the b* (yellow-blue axis) value exhibited an irregular trend, with a notable decrease for the combined treatment may be linked to a higher incidence of flavonoids, pigments often associated with a yellow hue [81]. This pattern aligns with findings from ultrasonicated germinated brown rice, where b* values decreased during germination due to enzymatic browning [82]. These variations observed across treatments suggests that the interaction between ultrasonication and germination parameters yields complex, non-linear effects on color development, influenced by balance between compounds extraction, mass transfer, and enzymatic oxidation [83].

**Table 2:** Effect of multi-frequency ultrasonication, soaking, germination and their combined treatments on CIELAB color coordinates and absorbance ratio of tartary buckwheat.

Processing treatments	Color coordinates				Absorbance ratio (%)	
	L*	a*	b*	ΔE	1047/1022	1022/995
Native	73.72 ± 0.23^ab^	1.14 ± 0.05^b^	11.57 ± 0.24^c^	−	82.31 ± 0.15^a^	92.02 ± 0.25^a^
MFUT	72.19 ± 0.12^c^	1.31 ± 0.03^a^	13.70 ± 0.04^a^	2.41 ± 0.08^b^	80.67 ± 0.21^b^	90.99 ± 0.21^b^
G	73.01 ± 0.18^abc^	1.10 ± 0.04^c^	11.45 ± 0.11^cd^	0.53 ± 0.17^c^	79.03 ± 0.12^c^	87.88 ± 0.22^d^
S	73.58 ± 0.19^a^	1.13 ± 0.06^b^	13.98 ± 0.08^a^	0.53 ± 0.17^c^	81.44 ± 0.15^ab^	92.43 ± 0.16^a^
MFUT + G	68.47 ± 0.58^d^	1.06 ± 0.10^d^	11.19 ± 0.02^b^	5.11 ± 0.58^a^	78.85 ± 0.23^d^	88.52 ± 0.013^cd^
MFUT + G + S	72.81 ± 0.32^b^	0.85 ± 0.05^e^	11.22 ± 0.12^d^	0.89 ± 0.18^c^	78.63 ± 0.24^d^	88.04 ± 0.22^c^

Native: Raw buckwheat; MUFT: multi-frequency ultrasonication; G: germination, S: soaking.

#### 3.7.2 Texture profile of native and treated *Fagopyrum tataricum*

**Fig. 5:**
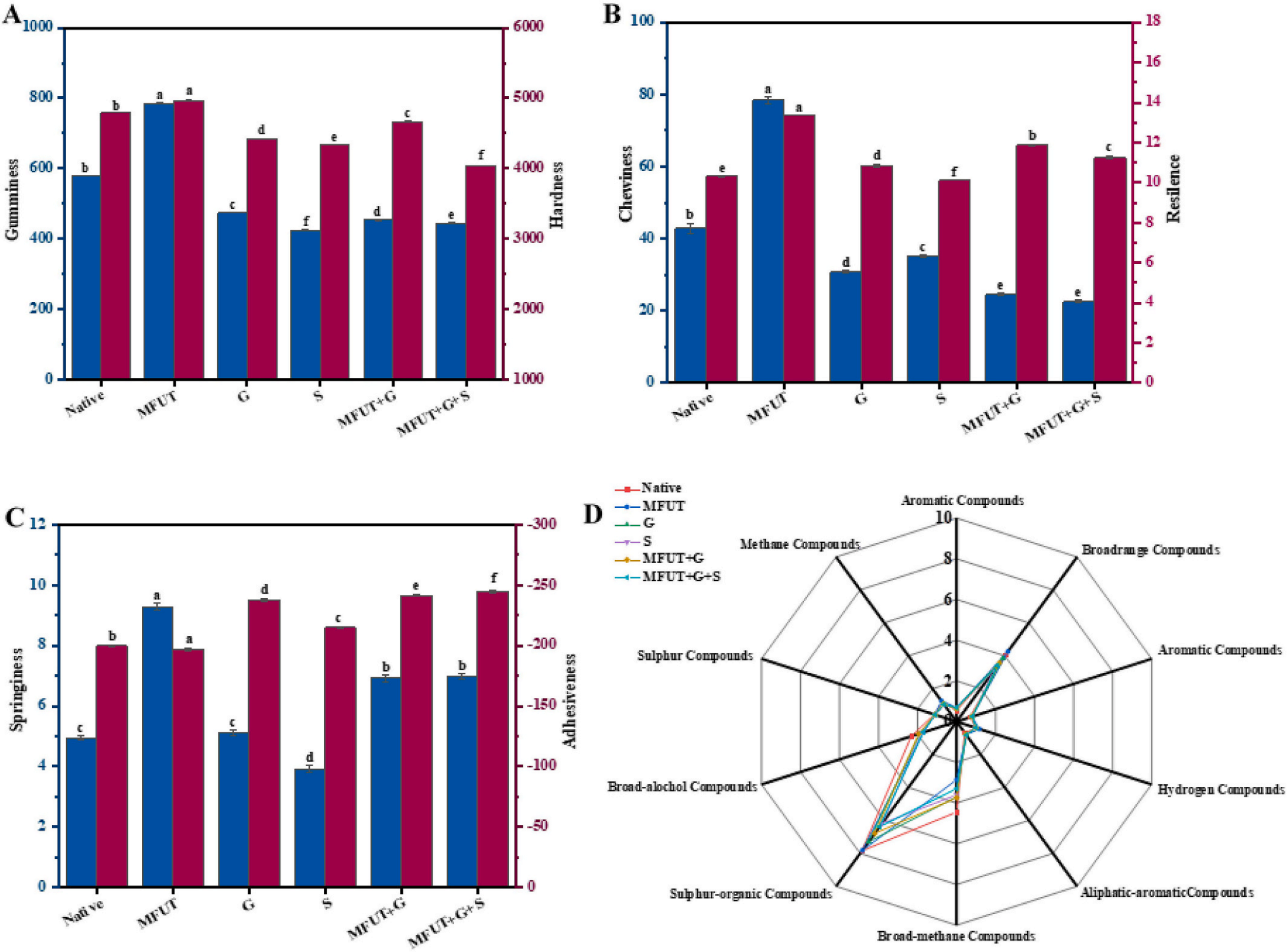
Impact of processing treatments on texture and aromatic profile of tartary buckwheat. A. Gumminess and hardness of treated tartary buckwheat. B. Chewiness and resilience of treated tartary buckwheat. C. Springiness and adhesiveness of treated tartary buckwheat. Radar plot of E-nose sensor array response, illustrating differences in volatile aromatic profile. Native: untreated tartary buckwheat, MFUT: multifrequency ultrasonication, G: germination, S: soaking.

Texture profile of *Fagopyrum tataricum* showed dynamic trends across all treatments compared to native group (Fig. 5A–C). MFUT treatment significantly (*p* < 0.05) increased hardness, resilience, cohesion, springiness, gumminess, and chewiness, mechanistically attributed to cavitation-induced cross linking, starch fragmentation, and enhanced water immobilization, strengthening dough network [84]. Conversely, G produced a softer matrix, with reduced textural parameters, likely due to starch and storage proteins hydrolysis, compromising structural integrity [85]. However, adhesiveness, the ability of a material to stick to a surface, followed an inverse trend; decreased after MFUT treatment due to compact structure and increased after germination, due to release of soluble carbohydrates, increasing stickiness. Contrary, the combined treatments (MFUT + G and MFUT + G + S) exhibited intermediate textural properties, indicating a functional balance between structural enforcement and enzymatic softening. This moderating effect offers versality for food formulations; firm-chewy profile of MFUT-treated TB, with its dense-elastic matrix aligns with textural requirements for plant-based meat analogues [86], whereas, the softened germinated matrix, with enhanced water retention, is well suited for porridges or slurry-based application [87]. This biphasic modulation is consistent with multi-frequency sono-germination studies in barley, which similarly demonstrate optimized hardness, gumminess, adhesion, and cohesion [88]. These findings underscore that texture can be selectively engineered in *Fagopyrum tataricum* flour through targeted pretreatments, yielding a functionally versatile ingredient for diverse food applications.

### 3.8 Structural characteristics of native and treated *Fagopyrum tataricum*

#### 3.8.1 FTIR spectroscopy of native and treated *Fagopyrum tataricum*

FTIR spectroscopy was used to examine the effect of multifrequency ultrasonication, germination, soaking and their combined treatments on structural modifications in *Fagopyrum tataricum* flour, presented in Fig. 4A, displaying characteristic absorption bands within the mid-infrared region. The FTIR spectra of all treatment groups exhibited a broad absorption band in 2900–3500 cm^−1^ region, characteristic of O–H and/or N–H vibrations [89]. The prominent peaks between 1650–1750 cm^−1^ region are ascribed to C=O carbonyl stretching, with components near 1650 cm^−1^ is specifically assigned to amide I protein associated C=O stretch [90]. Following ultrasonication, the O–H/N–H stretching band shifted marginally from 3289 cm⁻^1^ (native) to ∼ 3281 cm⁻^1^ in MFUT + G + S. this slight but consistent red shift suggests modest alterations in hydrogen bonding networks, likely due to disruption of intermolecular associations within polyphenol-protein matrix, as previously reported by Lou et al. [91]. There were no apparent absorption bands observed between 2000–2500 cm^−1^, ruling out the presence of triple bonds or cumulative double bonds (C≡C, C≡N) [17]. The absorption bands between 900–1200 cm^−1^ correspond to ordered crystalline regions, amorphous, and hydrated carbohydrates helix. The peak ratio 1047/1022 cm^−1^ reflects short-range order starch structure, with higher values reflecting greater molecular organization [92], decreased significantly across all treatments (Table 2). This decline indicates partial disruption of crystalline regions with greatest reduction observed in combined treatments MFUT + G + S (78.63 %) and MFUT + G (78.85 %), consistent with effects of starch processing [93]. Deconvolution of amide region (1600–1700 cm^−1^) further confirmed treatment-induced protein unfolding, as evidenced by a decrease in α-helix (10.97–10.25 %) and a concomitant increase in β-sheets (20.71–35.83 %) and random coils (10.74–13.31 %) (Fig. 4B). The MFIT + G + S treatment exhibited most pronounced effects, with a 6.56 % reduction in α-helix and 74.96 % and 23.92 % increase in β-sheets and random coils, respectively. These conformational changes are consistent with ultrasonication-induced destabilization of ordered protein structure [94], which appears to further enhanced by enzymatic activity during germination [95]and metabolic priming during soaking [96].

#### 3.8.2 XRD analysis of native and treated *Fagopyrum tataricum*

Starch exhibits a semi-crystalline organization arising from double-helical conformation of amylopectin, manifesting as characteristic XRD pattern presenting distinct peaks, however, physical and biochemical treatments can modify starch molecular configuration, thereby starch crystallinity [97]. XRD peaks and relative crystallinity (CI) for different treatment groups are presented in (Fig. 4C). Typically, cereal flours exhibit a characteristic A-type starch crystallinity pattern, with principal diffraction peaks at 15°, 17°, 18°, and 23° [31]. As evident from Fig. 4C all samples showed characteristic starch peaks with pronounced diffraction peaks at 15.31° and 23.19°, indicating that treatments didn’t alter the position of characteristic peaks, however, compared to native group the diffractograms of treated samples showed structural disordering, manifested by peak broadening and reduced peak intensities. The combined treatments MFUT + G + S and MFUT + G exhibited highest reduction in peak intensities indicating structural modifications associated with cavitational forces and enzymatic hydrolysis. Multi-frequency ultrasonication induced microstreaming shear indicates greater disruption of crystalline order by molecular rearrangement and local amorphization [98], whereas, activated endogenous enzymes such as α-amylase degrades starch (Fig. S2C), reducing crystalline regions, thereby increasing amorphous regions [99]. Furthermore, post-ultrasound soaking enhanced this phenomenon by facilitating hydration and metabolic activation. Relative crystallinity index (CI), denotes long-range order of starch computed as ratio of crystalline area to total area. All treatments significantly reduced indicating a reduction of 58.09 % and 40.77 % relative to native group, consistent with reduced peak intensities. Previous studies on ultrasonicated germinated oats [100] and pea [101] flours have also reported reduced peak intensities and crystallinity index.

#### 3.8.3 SEM of native and treated *Fagopyrum tataricum*

The morphological variations in *Fagopyrum tataricum* flour induced by various treatments are presented in Fig. 4E. The irregular clustered starch granule surfaces observed in native flour may be attributed to residual protein matrix and fiber components [102], consistent with aggregating effect reported for AOP-XG complexes [89], where similar surface clustering was associated with polysaccharide-protein interactions. Distinct morphological patterns emerged across treatments. MFUT-treated samples exhibited surface texturing with heterogeneous flake-like formations, consistent with cavitation-induced physical disruption reported in other ultrasonicated cereals [103], supporting the hypothesis that acoustic cavitation generates micro-scale mechanical stress on the flour matrix. Whereas germinated samples showed lower particulate disintegration with subtle surface modifications compared to the MFUT-treated sample, indicating enzymatic degradation. The morphology of soaked seed flours closely resembled that of the native sample, demonstrating minimal variation from hydration alone. Combined treatments (MFUT + G and MFUT + G + S) showed distinct morphologies compared with their individual treatments, exhibiting moderate particle integration and fewer surface asperities than MFUT-treated samples, suggesting potential implications for diverse processing behavior and functional performance [104]. The observed morphological changes are consistent with techno-functional characteristics: the textured surface in MFUT corresponds to higher WSI, whereas combined treatments consolidate morphology and explicates a distinct pasting profile.

### 3.9 Thermal properties of native and treated *Fagopyrum tataricum*

Starch gelatinization is a thermal transition wherein crystalline domains undergoes irreversible disruption upon heating in presence of water, transitioning from a semi-crystalline to an amorphous state. Gelatinization enthalpy (ΔH) quantifies heat energy required for starch transition, reflecting the degree of ordered molecules [105]. Gelatinization temperature including onset temperature (To), peak temperature (Tp) and conclusion temperature (Tc) are presented in Table 3. The thermal transition temperature showed an increasing trend across all treatment groups except for G group, exhibiting an endothermic transition between 60 °C and 74 °C, corresponding primarily to starch gelatinization, with potential overlap from protein denaturation (Xing et al., 2021; López et al., 2018).The reduction in gelatinization temperature of germinated group could be associated α-amylase mediated starch depolymerization leading to rapid gelatinization [107], whereas, increased transition temperature for MFUT group may ascribed to molecular reaggregation, generating partially ordered structure requiring higher temperature for gelatinization [108]. To and Tc indicates the disruption of weak bonds and strong bonds, increased from 60.19 °C and 71.45 °C (control) to 64.53 °C and 73.85 °C (MFUT + G + S) and 65.15 °C and 74.23 °C (MFUT + G). Whereas, Tp represents maximum energy absorption for starch gelatinization, serving as an index of molecular order, also increased from 66.23 °C (control) to 72.68 °C (MFUT + G) and 71.34 °C (MFUT + G + S). However, ΔH decreased (7.51––3.15 J/g) significantly across all the treatments groups relative to control, confirming ultrasonication and germination induced starch weaking and structural damage, corroborated by our findings (Fig. 4C) and aligned with previous studies [109]. The observed divergent gelatinization patterns reflect variations and starch composition and processing conditions, which governs the balance between crystalline disruption and molecular recrystallization [110], [111].

**Table 3:** Effect of multi-frequency ultrasonication, soaking, germination and their combined treatments on thermal properties of tartary buckwheat.

Processing treatments	T_o_ (℃)	T_p_ (℃)	T_c_ (℃)	T_C_ −T_O_ (℃)	ΔH (J/g)	PV (cP)	TV (cP)
**Native**	60.19 ± 0.05^d^	66.23 ± 0.15^d^	71.45 ± 0.05^d^	11.26 ± 0.12^a^	7.51 ± 0.03^c^	3275 ± 0.42^a^	3089 ± 0.57^a^
**MFUT**	62.20 ± 0.04^c^	70.24 ± 0.14^c^	73.05 ± 0.14^b^	10.85 ± 0.05^b^	5.65 ± 0.03^c^	2570 ± 0.57^b^	2502 ± 0.14^b^
**G**	59.75 ± 0.19^e^	65.83 ± 0.09^e^	70.98 ± 0.06^e^	11.23 ± 0.06^ab^	6.23 ± 0.02^b^	2461 ± 0.71^c^	2336 ± 0.25^d^
**S**	60.32 ± 0.12^d^	66.46 ± 0.07^d^	71.15 ± 0.07^d^	10.83 ± 0.08^b^	7.35 ± 0.04^a^	2596 ± 0.28^ab^	2485 ± 0.41^c^
**MFUT + G**	65.15 ± 0.07^a^	72.68 ± 0.04^a^	74.23 ± 0.05^a^	8.70 ± 0.17^c^	4.02 ± 0.02^d^	2005 ± 0.85^e^	1928 ± 0.84^f^
**MFUT + G + S**	64.53 ± 0.04^b^	71.34 ± 0.04^a^	73.85 ± 0.05^a^	11.54 ± 0.12^a^	3.15 ± 0.24^e^	2371 ± 0.14^d^	2198 ± 0.69^e^

Native: Raw buckwheat; MUFT: multi-frequency ultrasound treatment; G: germination, S: soaking.

### 3.10 Pasting properties of native and treated *Fagopyrum tataricum*

The pasting properties of *Fagopyrum tataricum* are presented in Fig. 4D with corresponding parameters summarized in Table S2, demonstrating a decreasing trend across all parameters (peak viscosity, trough viscosity, final viscosity, breakdown viscosity, and setback viscosity). Ultrasonication induced mechanical disruption of granular starch structure via intense shear microjets from subsequent bubbles collapse [112], causing starch polymer fragmentation and surface pitting, as evidenced by SEM (Fig. 4E). Whereas, germination-driven starch and protein hydrolysis into smaller subunits, further reduce structural order [113]. Peak viscosity (PV) is a measure of starch swelling capacity, resulting from gelatinization induced disintegration of granular network. PV reduced across all treatment with highest reduction in MFUT + G (3275–2005 cP) and MFUT + G + S (3275–2371 cP), could be attributed to ultrasound induced structural damage increased substrate susceptibility to germination activated enzymes.

Breakdown viscosity (BV) trends reflects susceptibility of swollen starch granules to disintegration under shear and heating, whereas, set back viscosity (SV), denotes starch retrogradation tendency, driven by reassociation of amylose chains upon cooling. Both parameters exhibited pronounced reductions, indicating progressive structural destabilization and diminished retrogradation potential. BV and SV decreased significantly (*p* < 0.05) in MFUT + G (77 cP and 1413 cP) followed by MFUT (68 and 2761 cP) and G (125 and 2343 cP), compared to control (186 cP and 4016 cP). These trends reflect the combined effect of cavitation-mediated crystalline disruption and enzymatic hydrolysis, composing granular integrity and molecular ordering. Notably, both BV (77 cP) and SV (173 cP) recovered in MFUT + G + S treatment, may be attributed to annealing effect during post-ultrasound soaking phase, where in controlled hydration may facilitate molecular reassociation, restoring granular stability and retrogradation [12]. The partial recovery of PV, BV and SV in MFUT + G + S compared to MFUT + G, despite having lowest ΔH (3.15 J/g), peculiar behavior may indicate retrogradation or molecular association during sequential processing, where degraded starch chains reorganized into ordered structures upon cooling, partially recovering viscosity [114]. These findings demonstrate that combined ultrasonication and germination effectively modulates pasting behavior, suitable for gluten-free bakery, extruded snacks, and weaning foods requiring controlled texture and stability [115].

### 3.11 Aromatic profile of native and treated *Fagopyrum tataricum*

E-nose was employed to evaluate the aroma profiles of *Fagopyrum tataricum* flour samples, showed an indicated substantial sensor response shift across MFUT, G and combined treatment groups (Fig. 5D), confirmed by distinct clustering in Principal component analysis (PCA), with PC1-PC3 accounting for >99 % of total variance (Fig. S3). The most pronounced response was observed for W3C (aromatic compounds), W1W (sulfur-organic compounds), W1S (broadrange methane compounds), and W2S (broadrange compounds) sensors ranging between, 0.67–0.81, 7.84–6.38, 4.45–3.29, 4.63–3.40, respectively for MFUT + G + S. These observed response patterns likely reflect the activation of endogenous enzymes during germination and soaking, converting lipids and amino-acids into volatile compounds while simultaneously degrading astringent. Additionally, ultrasonication may facilitates the release of bound aromatics, contributing to the altered sensor response profiles, suggesting additive enhancement of aromatic intensity and complexity, that should be further investigated by individual compound profiling through GC–MS. These findings are consistent with studies across diverse plant matrices, which report that ultrasonication, germination, and their combinations can modify volatile profiles [36], [88], [116].

## 4 Conclusion

The present study conclusively demonstrates that sequential application of multifrequency ultrasonication, soaking and germination (MFUT + G + S) effectively enhanced the bioactive potential, structural integrity, and techno-functional properties of *Fagopyrum tataricum* flour. The treatment maximized the antioxidant enzyme activities, reduced anti-nutrients, and improved protein digestibility, while structural modification such as reduced starch crystallinity, particle size and protein unfolding translated into superior hydration, emulsifying, textural and thermal properties and aroma profile. These findings suggest that MFUT + G + S treatment offers a promising green processing strategy for producing functionally enhanced flour. Future studies should focus on investigating *in-vivo* nutritional bioavailability and sensory acceptability of this flour for targeted food formulations such as gluten-free food products and their industrial scalability.

## CRediT authorship contribution statement

**Zunaira Basharat:** Writing – original draft, Methodology, Formal analysis, Data curation, Conceptualization. **Bin Dai:** Writing – review & editing, Formal analysis, Data curation. **Huicong Xu:** Methodology, Formal analysis, Data curation. **JiaYu Yin:** Investigation, Formal analysis, Data curation. **Aysha Imtiaz:** Writing – review & editing, Visualization, Software, Methodology, Conceptualization. **Muhammad Safiullah Virk:** Writing – review & editing, Data curation. **Sanabil Yaqoob:** Writing – review & editing, Validation, Software, Data curation, Conceptualization. **Yuqing Duan:** Writing – review & editing, Supervision, Resources, Conceptualization. **Kai Hu:** Writing – review & editing, Formal analysis, Data curation. **Meihong Cai:** Writing – review & editing, Software, Methodology. **Haihui Zhang:** Writing – review & editing, Supervision, Resources, Project administration, Conceptualization. **Qing Shen:** Writing – review & editing, Project administration, Methodology, Funding acquisition, Conceptualization. **Chunlai Zeng:** Writing – review & editing, Supervision, Project administration, Funding acquisition, Conceptualization.

## Declaration of competing interest

The authors declare that they have no known competing financial interests or personal relationships that could have appeared to influence the work reported in this paper.
