# Supplementary material for: Structural and functional modulation of Fagopyrum tataricum by multifrequency ultrasonication, germination and soaking: impact on polyphenols and techno-functional properties
Source: Ultrason Sonochem. 2026 Aug 5;132:107994. doi: 10.1016/j.ultsonch.2026.107994 (PMC13476772; doi:10.1016/j.ultsonch.2026.107994)
Supplement: Supplementary file 1 [file mmc1.docx]

Figure S1: Single-factor optimization of germination duration (a-b), multi-frequency ultrasonication duration (c-d), and soaking duration (e-f) based on responses for bioactive compounds and anti-nutritional factors.

**(a)**

**(b)**

**(c)**

Figure S2: Germination efficiency of tartary buckwheat processed under various processing treatments. (a). Germination rate of tartary buckwheat. (b). α-amylase activity of tartary buckwheat at respective germination time. (c). α-amylase activity of treated tartary buckwheat.

Figure S3: Principal Component Analysis (PCA) biplot of E-nose data, indicating samples clustering and contribution of individual sensors.

**Table S1: Leaching effect of soaking time on soaked tartary buckwheat**

| **Soaking Time (hrs)** | **Variables** | | | |
| --- | --- | --- | --- | --- |
|  | pH | TPC (mg/L) | Tannins (mg/L) | Phytic acid (mg/L) |
| 0 | 6.685±0.04^a^ | - | - | - |
| 3 | 3.42±0.16^c^ | 9.77±0.67^b^ | 19±0.35^b^ | 0.407±0.01^b^ |
| 6 | 5.635±0.07^b^ | 17.19±0.79^a^ | 26.08±1.20^a^ | 0.675±0.03^a^ |

| **Processing Treatments** |  | **PV (cP)** | **TV (cP)** | **BV (cP)** | **FV (cP)** | **SV (cP)** |
| --- | --- | --- | --- | --- | --- | --- |
| **Native** |  | 3275±0.42^a^ | 3089±0.57^a^ | 186±0.57^a^ | 7105±0.42^a^ | 4016±0.14^a^ |
| **MFUT** |  | 2570±0.57^b^ | 2502±0.14^b^ | 68±0.14^e^ | 5263±0.85^b^ | 2761±0.71^c^ |
| **G** |  | 2461±0.71^c^ | 2336±0.25^d^ | 125±0.28^c^ | 4679±0.71^d^ | 2343±0.42^d^ |
| **S** |  | 2596±0.28^ab^ | 2485±0.41^c^ | 111±0.42^cd^ | 5575±0.28^ab^ | 3090±0.14^ab^ |
| **MFUT+G** |  | 2005±0.85^e^ | 1928±0.84^f^ | 77±0.85^d^ | 3341±0.57^e^ | 1413±0.28^e^ |
| **MFUT+G+S** |  | 2371±0.14^d^ | 2198±0.69^e^ | 173±0.71^b^ | 5206±0.35^c^ | 3008±0.35^b^ |

**Table S2: Effect of multi-frequency ultrasonication, soaking, germination and their combined treatments on pasting properties of tartary buckwheat.**

Native: Raw buckwheat; MUFT: multi-frequency ultrasound treatment; G: germination, S: soaking
